# Man versteht das Heute nur, wenn man auch das Gestern kennt: Geschichte der Rhythmologie in Deutschland

**DOI:** 10.1007/s00399-024-01013-1

**Published:** 2024-03-08

**Authors:** M. Borggrefe, F. de Haan, C. W. Israel

**Affiliations:** 1 Universitätsmedizin Mannheim, I. Medizinische Klinik, Mannheim, 68167 Theodor-Kutzer-Ufer 1-3, Deutschland; 2 Historisches Archiv, Deutsche Gesellschaft für Kardiologie – für Herz und Kreisllaufforschung e.V., Düsseldorf, 40237 Grafenberger Allee 100, Deutschland; 3 Klinik für Innere Medizin – Kardiologie und Angiologie, in Bethel/Haus Gilead I, Evangelisches Krankenhaus Bielefeld, Burgsteig 13, 33617 Bielefeld, Deutschland

Seit dem Mittelalter haben Störungen des regelmäßigen Herzschlags, bedingt durch eine oft dramatische Symptomatik, die Ärzte beschäftigt, ja alarmiert. Erst mit der Erfindung des Elektrokardiogramms durch A. Waller und W. Einthoven Ende des 19. Jahrhunderts war aber eine therapeutische Option durch Zuordnung der Rhythmusstörung überhaupt möglich.

In den ersten Jahrzehnten des 20. Jahrhunderts standen vor allem die bradykarden Rhythmusstörungen im Fokus des Interesses, und es gelang, mit Hilfe namhafter Pathologen und Physiologen tiefere Erkenntnisse über SA-, AV- und Schenkelblockierungen zu gewinnen, die dann am 06.10.1961 zur ersten erfolgreichen Herzschrittmacherimplantation in Deutschland führten.

Invasive elektrophysiologische Untersuchungen bestanden Mitte der 1960er-Jahre in der Ableitung und Messung der HV-Zeit.

Anfang der 1970er-Jahre wurde die programmierte Stimulation eingeführt und das Interesse galt der Erforschung von Tachykardiemechanismen.

Die erste Implantation von Defibrillatoren in Deutschland 1984 eröffnete eine innovative Therapieoption in der nichtpharmakologischen Behandlung ventrikulärer Arrhythmien. Die kurative Behandlung von Tachykardien wurde initial gemeinsam von Herzchirurgen und Elektrophysiologen durchgeführt. Durch die Einführung der Hochfrequenzablation wurde die Rhythmus-Chirurgie am offenen Herzen vollständig abgelöst. Heute ist die Katheterablation Mittel der Wahl in der nichtpharmakologischen Therapie von Tachykardien jeglichen Ursprungs.

Basierend auf den Bad Nauheimer EKG-/Arrhythmiekursen von A. Weber wurde am 03.06.1927 die Deutsche Gesellschaft für Kardiologie von B. Kisch u. a. gegründet. Daher erscheint es jetzt – fast 100 Jahre später – angemessen und sinnvoll, über die historische Entwicklung der Diagnostik und Therapie von Herzrhythmusstörungen nachzudenken und zu resümieren.

Das vorliegende Sonderheft der Zeitschrift *Herzschrittmachertherapie und Elektrophysiologie* stellt diese Entwicklung in einzigartiger Weise und mit einzigartigen Autoren vor! Von den Anfängen des Verständnisses von EKG und invasiver Elektrophysiologie über die Entwicklung der Device-Therapie von Herzschrittmachern bis zur kardialen Resynchronisationstherapie mit und ohne Defibrillator-Back-up, schließlich zur Katheterablation mit dreidimensionalem Mapping und dem genetischen Hintergrund von Arrhythmien, wird die Geschichte der Rhythmologie in Deutschland in faszinierender Weise dargestellt. An dieser Stelle danken wir allen beteiligten Autoren für die hervorragenden und fundierten Beiträge! Unser Dank gilt ebenso den Sponsoren, die eine Publikation dieses Sonderheftes erst möglich gemacht haben.

Man versteht das Heute nur, wenn man auch das Gestern kennt. In diesem Sinne wünschen wir allen Lesern viel Spaß und Erkenntnisgewinn bei der Lektüre!

M. Borggrefe

F. de Haan

C. W. Israel

## Interessenkonflikt

C. W. Israel: Beraterhonorar: Impulse-Dynamics, Medtronic, Zoll; Honorare für Vorträge: Abbott, Biotronik, Boston-Scientific, Impulse-Dynamics, Medtronic, MicroPort, Zoll; Erstattung von Reisekosten und Kongressgebühren: Abbott, Biotronik, Boston-Scientific, Impulse-Dynamics, Medtronic, MicroPort, Zoll. M. Borggrefe und F. de Haan geben an, dass kein Interessenkonflikt vorliegt.

The supplement containing this article is sponsored by Abbott, Biosense Webster, Biotronik, Boston Scientific, Deutsche Herzstiftung, Medtronic, MicroPort, OSYPKA and ZOLL.

